# Association between Estrogen Receptor Alpha Gene Polymorphisms and Susceptibility to Idiopathic Scoliosis in Bulgarian Patients: A Case-Control Study

**DOI:** 10.3889/oamjms.2015.054

**Published:** 2015-05-20

**Authors:** Svetla Nikolova, Vasil Yablanski, Evgeni Vlaev, Luben Stokov, Alexey Savov, Ivo Kremensky

**Affiliations:** 1 *Medical University-Sofia, National Genetic Laboratory, Sofia, Bulgaria*; 2 *Tokuda Hospital Sofia, Orthopedic and Traumatology Clinic, Sofia, Bulgaria*; 3 *University Orthopedic Hospital “Prof. Boycho Boychev”, Orthopedic and Traumatology, Sofia, Bulgaria*

**Keywords:** Idiopathic Scoliosis, Estrogen receptor alpha gene, Polymorphisms, Susceptibility, Progression

## Abstract

**BACKGROUND:**

The current consensus on idiopathic scoliosis maintains that it has a multifactorial etiology with genetic predisposing factors.

**AIM:**

Estrogen receptor alpha gene has been considered as candidate gene of idiopathic scoliosis.

**MATERIAL AND METHODS:**

We conducted a case-control study of Bulgarian population samples (eighty patients with idiopathic scoliosis and one hundred-sixty healthy unrelated gender-matched controls) trying to investigate the association between common genetic polymorphisms of estrogen receptor alpha and the susceptibility to idiopathic scoliosis. Molecular detection of the restriction polymorphisms XbaI and PvuII was performed by polymerase chain reaction following by restriction fragment length polymorphism. The statistical analysis was performed by Pearson’s chi-squared test.

**RESULTS:**

Our case-control study showed statistically significant association between the PvuII polymorphism and susceptibility to idiopathic scoliosis and curve progression. No genotype or allele of XbaI polymorphism was found to be correlated with the onset or severity of the disease.

**CONCLUSIONS:**

The identification of molecular markers with diagnostic and prognostic value could be useful for early detection of children at risk for the development of scoliosis and for prognosis of the risk for a rapid deformity progression. That would facilitate the therapy decisions and early stage treatment of the patient with the least invasive procedures.

## Introduction

Since its first documentation by Hippocrates, the diagnosis, cause and treatment of idiopathic scoliosis (IS) have been the focus of a great deal of research and yet the etiology remains enigmatic [1]. The current consensus on IS maintains that it has a multifactorial etiology with genetic predisposing factors. The study on genetics of IS having indicated substantial genetic heterogeneity in the etiology of the disease.

In Europe, during the period from 2002 to 2014 common polymorphisms in 13 candidate-genes for IS [2-10] have been investigated. Two studies on gene expression have been performed and several genes with change expression in patients with IS have been reported [11, 12].

The *estrogen receptor alpha gene* (*ESR1*) has been considered as candidate gene of IS [7, 13]. *ESR1* gene was shown to be expressed in both human osteoblasts and osteoclasts [14] and mutations of the *ESR1* gene were shown to cause bone loss and delayed skeletal growth in affected humans [15]. The specific XbaI and PvuII polymorphisms of the *ESR1* gene were associated with low bone mineral density (bMD) at all bMD measurement sites in a Bulgarian female population sample [16].

Associations between IS and common single nucleotide polymorphisms (SNPs) in *ESR1* have been reported. A study conducted by Inoue et al. in 2002 was the first one which investigated the role of the *ESR1* in the pathogenesis of IS and concluded that the *ESR1* XbaI polymorphism constitutes important factor for the curve progression in Japanese population [13]. In 2006 the XbaI site polymorphism of *ESR1* gene was associated with a risk of IS in Chinese population [17]. A replication study conducted by Tang et al. revealed no association between the two *ESR1* SNPs and the occurrence of IS or curve severity in Chinese population [18]. A Chinese study conducted by Zhao et al. in 2009 indicated there are statistically significant differences between patients with double curve, with Cobb angle ≥ 40° and with thoracic curve and healthy controls in the polymorphic distribution of the PvuII polymorphism of the *ESR1* [19].

In 2011 a replication study conducted by Takahashi et al. found no association of XbaI and PvuII polymorphisms with IS predisposition or curve severity in the Japanese population [20]. No association was found between the *ESR1* restriction polymorphisms and IS in Poland females [7, 8].

In the present study the association of the XbaI and PvuII common polymorphisms of *ESR1* with susceptibility to IS was investigated in Bulgarian patients.

## Material and Methods

In this study, patients with IS (n = 80) and healthy unrelated gender-matched controls (n = 160) were included. All participants in the study were informed about its purpose and were included only after the subjects/families signed their informed consent. Peripheral blood samples were obtained from patients and control subjects. The study protocol was approved by the Ethics Committee of the Medical University-Sofia (No 2987/2012).

### Patients

From 2012-2014, patients with IS were recruited with the help of orthopaedic surgeons from Tokuda Hospital Sofia and University Orthopedic Hospital “Prof. B. Boychev”. The IS diagnosis was confirmed clinically and radiologically. The curves were measured by the Cobb method. The Cobb angle under consideration was either the final Cobb angle, defined as the curve angle in skeletally mature patients (n = 35) or Cobb angle, measured in the last follow up of those patients who were skeletally immature (n = 45). The average Cobb angle was 52.7° (range of 16° to 100°). The average age at the beginning of the disease was 11.2 years (range of 2 to 15 years). In this study, male (n = 15) and female (n = 65) patients were included.

IS was observed in three age groups: infantile - from one to three years of age (n = 3), juvenile - from greater than three years of age through nine years of age (n = 16) and, adolescent from ten to sixteen years of age (n = 61). Scoliosis as a phenotypic characteristic like Marfan’s syndrome was excluded.

### Controls

The control group including healthy subjects without clinical signs of IS was recruited from a pool of unrelated gender-matched volunteers from other Units and Clinics of Tokuda Hospital Sofia, National Genetic Laboratory, hospital staff members and students. The controls were selected among adult patients with skeletal maturity with negative family history of IS. Radiological examination was not performed in the control group.

### Genotyping

Genomic DNA was extracted from the peripheral blood leucocytes using magnetic bead technology (chemagic DNA Blood Kit special, Chemagen) on automated high throughput nucleic acid isolation platform (chemagic Magnetic Separation Module I, Chemagen).

The polymorphic region of the *ESR1* was amplified by polymerase chain reaction (PCR) with the following primer set: for-5’-CTGCCACCCTATCTGTATCTTTTCCTATTCTCC-3’ and rev-5’-TCTTTCTCTGCCACCCTGGCGTCGATTATCTGA-3’ [7, 13].

The PCR was carried out in a a reaction mix of 20µl containing 100-ng DNA and 10X Prime Taq buffer (Genet Bio), 10 mM dNTPs Mixture (Genet Bio), 20 pmol Forward and Reverse primers (AlphaDNA), and 0.1 U Prime Taq DNA Polymerase (Genet Bio). PCR ampifications were performed in an AB 2720 Thermocycler (Life Technologies) with an initial denaturation at 94°C for ten minutes and a final extension of ten minutes at 72°C. The following thermal cycle was repeated 30 times: denaturation at 94°C for 60 seconds, annealing for 60 seconds at 59°C, and extension at 72°C for 120 seconds.

The PCR products were cleaved with the appropriate restriction enzymes (New England Biolabs), according to the manufacturer’s instructions, and the digested products were separated on agarose 3% gel in VG-SYS Horizontal Electrophoresis System (Biochrom). The restriction enzymes and the lengths of the fragments representing the genotypes of the genes are presented in Table 1.

**Table 1:** RFLP protocol

Gene, Polymorphism	PCR Product Size, bp	Restriction Enzyme	Restriction Fragments, bp
***ESR1 rs9340799***	1374	XbaI	XX: 1374

			Xx: 1374+982+392

			xx: 982+392

***ESR1 rs2234693***	1374	PvuII	PP: 1374

			Pp: 1374+937+437

			pp: 937+437

### Statistical analysis

The *ESR1* genotypes were compared using χ2 test with a *P* value of 0.05 as statistically significant. All calculations were performed with the statistical program SPSS 19.0 software package for Windows.

## Results

This study examined the association between IS and two SNPs: the XbaI (rs9340799) and the PvuII (rs2234693) restriction polymorphisms of *ESR1*. Genotypes were in Hardy-Weinberg equilibrium.

The overall frequency of the PP genotype of the *ESR1* PvuII polymorphism in the patients with IS was two times higher than in the controls (32.5% vs. 16.9%) and the frequency of the P allele of the *ESR1* PvuII polymorphism in the patients with IS was also higher than in the controls (57.5 % vs. 47.8 %). In conclusion, the homozygous PP genotype of *ESR1* was associated with a higher risk of scoliosis (PP vs. Pp+pp, p=0.006; OR: 2.37; 95% CI: 1.27 – 4.43) and the presence of the P allele alone (P vs. p, p = 0.045; OR: 1.48, CI: 1.01 - 2.17) could be considered as susceptibility factor to IS.

The overall frequency of the XX genotype of the *ESR1* XbaI polymorphism in the patients with IS was higher than in the controls (22.5% vs. 13.8%) and the frequency of the X allele of the *ESR1* XbaI polymorphism in the patients with IS was also higher than in the controls (49.4 % vs. 41.9 %) but no significant association was detected (p>0.05). In conclusion, the presence of the X allele alone and the homozygous XX genotype of *ESR1* could not be considered as susceptibility factor to IS.

There were no statistically significant differences in the genotype frequencies of PvuII polymorphism (33.3% vs. 37.5% vs. 31.1%, p=0.2) and XbaI polymorphism (33.3% vs. 25.0% vs. 21.3%, p=0.67) of *ESR1* in the different age groups. The SNPs of *ESR1* were not associated with the age of onset of IS in Bulgarian patients.

In the surgical treatment group (n=62) where Cobb angle>40° the frequency of the PP genotype of *ESR1* was significantly higher than in the controls (p=0.023) and increased OR was observed (OR=2.18; CI: 1.1-4.3). The XX genotype and the X allele were not significantly associated with curve severity (p=0.061; OR: 2.0; 95% CI: 0.96-4.18).

In the small group of male patients (n = 15) no significant associations were found (p > 0.05). In the group of female patients (n = 65) the frequencies of the PP genotype and the P allele of *ESR1* were significantly higher than in the female controls (p = 0.004 and p = 0.032, respectively). The XX genotype and the X allele were not significantly associated with gender (p > 0.05).

The p-values and odds ratios of genotypes and alleles are summarised in Table 2.

**Table 2:** Odds ratios of genotypes and alleles in the different subgroups

Subgroup	Genotype, Allele	p-value	OR [95% CI]
**General group**	PP vs. Pp + pp P vs. p	**0.006 0.045**	2.37 [1.27-4.43] 1.48 [1.01-2.17]

	XX vs. Xx + xx X vs. x	0.086 0.12	1.82 [0.91-3.64] 1.35 [0.92-1.98]

**Cobb angle >40^0^**	PP vs. Pp + pp P vs. p	**0.023** 0.24	2.18 [1.1-4.3] 1.28 [0.85-1.94]

	XX vs. Xx + xx X vs. x	0.061 0.12	2.0 [0.96-4.18] 1.39 [0.92-2.1]

**Males**	PP vs. Pp + pp P vs. p	1 0.89	1.25 [0.26-6.12] 1.07 [0.44-2.57]

	XX vs. Xx + xx X vs. x	0.65 1	2.25 [0.4-12.8] 1.0 [0.42-2.41]

**Females**	PP vs. Pp + pp P vs. p	**0.004 0.032**	2.69 [1.36-5.33] 1.59 [1.04-2.44]

	XX vs. Xx + xx X vs. x	0.14 0.083	1.75 [0.82-3.73] 1.45 [0.95-2.22]

We also investigated the combinatorial effect of these polymorphisms on risk of IS. The analysis of all possible two-way polymorphism combinations revealed significant associations between two genotype combinations and genetic predisposition to IS and curve severity (Table 3).

**Table 3:** Odds ratios of genotype combinations XbaI-PvuII

	Genotype Combinations	OR [95% CI]	p-value
**General group**	XX-Pp	4.94 [1.47-16.59]	0.007

	Xx-PP	4.38 [1.77-10.85]	0.001

**Cobb angle >40^0^**	XX-Pp	4.18 [1.14-15.36]	0.031

	Xx-PP	3.23 [1.18-8.79]	0.024

## Discussion

Predisposition for IS, like other examples of complex traits, does not have a specific assigned risk of heritability, but inheritance is based on multiple factors, potentially both genetic and environmental [21].

We examined previously reported genetic associations [7, 8, 13, 17-20] between IS and two SNPs: XbaI and PvuII restriction polymorphisms of *ESR1* among Bulgarian patients.

In our study, the frequencies of the genotypes of PvuII polymorphism of *ESR1* showed statistically significant differences between cases and controls (χ^2^ = 7.57, p = 0.023) and the PP genotype of *ESR1* could be considered as a predisposing factor for IS (PP vs. Pp+pp, p = 0.006; OR: 2.37; 95% CI: 1.27– 4.43). The P allele was associated with higher risk for development of IS (P vs. p, p = 0.045; OR: 1.48, CI: 1.01-2.17).

The genotype and allele frequencies of the XbaI polymorphism of *ESR1* were comparable between cases and controls (χ^2^=5.084; p = 0.079) and the genotypes and alleles alone could not be considered as a susceptibility factor of IS.

In the subgroup of surgical cases where Cobb angle >40° a significant association between the PP genotype of *ESR1* and IS was detected. The homozygous PP genotype of *ESR1* could possess a modifying effect on the pathological phenotype. At the same time no statistically significant association between XbaI polymorphism and severity of the disease was found among Bulgarian patients.

In the subgroup of female patients a statistically significant association between the *ESR1* PvuII polymorphism and the clinical phenotype was observed. The XbaI polymorphism was not associated with the gender.

In this way, a previously reported positive genetic association between PvuII polymorphism and the risk of rapid progression of IS in Chinese patients [19] was confirmed among Bulgarian patients. The previously reported positive associations between XbaI polymorphism and curve progression [13, 17] were not established but the negative associations from the replication studies in Asian populations [18, 20] were confirmed in Bulgarian patients.

Janusz et al. found no difference either in XbaI (p = 0.87) or PvuII (p = 0.36) allele distribution between Poland female patients with IS and healthy gender-matched adult controls [7, 8]. The observed differences could be explained with the differences of the genotype and allele frequencies within the different populations and ethnic groups.

We also investigated the combinatorial effect of these polymorphic variants on risk of IS. The genotype combinations of XX-Pp and Xx-PP of *ESR1* showed significantly elevated ORs (4.94 and 4.38, respectively). The hypothesis for synergistic effect of SNPs on the etiology and progression of IS has been widely accepted and reported [3].

In conclusion, this case-control study revealed statistically significant association between the *ESR1* PvuII genetic polymorphism and the susceptibility to IS and curve severity among Bulgarian patients.

Our results suggest that the identification of molecular markers with diagnostic and prognostic value could be useful for early detection of children at risk for the development of IS and for prognosis of the risk for a rapid deformity progression. That would facilitate the therapy decisions and early stage treatment of the patient with the least invasive procedures.

## References

[1] Gorman KF, Julien C, Oliazadeh N, Tang Q, Moreau A (2012). The genetic epidemiology of idiopathic scoliosis. Eur Spine J.

[2] Aulisa L, Papaleo P, Pola E, Galli M, Aulisa L (2007). Association between IL-6 and MMP-3 gene polymorphisms and adolescent idiopathic scoliosis:a case-control study. Spine.

[3] Morocz M, Czibula A, Grozer ZB (2011). Association study of BMP4, IL6, Leptin, MMP3, and MTNR1B gene promoter polymorphisms and adolescent idiopathic scoliosis. Spine.

[4] Yilmaz H, Zateri C, Uludag A, Bakar C, Kosar S, Ozdemir O (2012). Single-nucleotide polymorphism in Turkish patients with adolescent idiopathic scoliosis:Curve progression is not related with MATN-1, LCT C/T-13910, and VDR BsmI. Journal of Orthopaedic Research.

[5] Ryzhkov II, Borzilov EE, Churnosov MI, Ataman AV, Dedkov AA, Polonikov AV (2013). Transforming growth factor beta 1 is a novel susceptibility gene for adolescent idiopathic scoliosis. Spine.

[6] Esposito T, Uccello R, Caliendo R (2009). Estrogen receptor polymorphism, estrogen content and idiopathic scoliosis in human:a possible genetic linkage. J Steroid Biochem Mol Biol.

[7] Janusz P, Kotwicki T, Andrusiewicz M, Kotwicka M (2013). XbaI and PvuII polymorphisms of estrogen receptor 1 gene in females with idiopathic scoliosis:no association with occurrence or clinical form. PLoS One.

[8] Janusz P, Kotwicka M, Andrusiewicz M, Czaprowski D, Czubak J, Kotwicki T (2014). Estrogen receptors genes polymorphisms and age at menarche in idiopathic scoliosis. BMC Musculoskelet Disord.

[9] Montanaro L, Parisini P, Greggi T (2006). Evidence of a linkage between matrilin-1 gene (MATN1) and idiopathic scoliosis. Scoliosis.

[10] Zorkoltseva IV, Liubinskiı OA, Sharipov RN, Zaidman AM, Aksenovich TI, Dymshits GM (2002). Analysis of polymorphism of the number of tandem repeats in the aggrecan gene exon G3 in the families with idiopathic scoliosis. Russ J Genet.

[11] Nowak R, Szota J, Mazurek U (2012). Vitamin D receptor gene (VDR) transcripts in bone, cartilage, muscles and blood and microarray analysis of vitamin D responsive genes expression in paravertebral muscles of juvenile and adolescent idiopathic scoliosis patients. BMC Musculoskelet Disord.

[12] Nowak R, Kwiecien M, Tkacz M, Mazurek U (2014). Transforming Growth Factor-Beta (TGF-β) Signaling in Paravertebral Muscles in Juvenile and Adolescent Idiopathic Scoliosis. BioMed Research International.

[13] Inoue M, Minami S, Nakata Y (2002). Association between Estrogen Receptor Gene Polymorphisms and Curve Severity of Idiopathic Scoliosis. Spine.

[14] Pensler JM, Radosevich JA, Higbee R, Langman CB (1990). Osteoclasts isolated from membranous bone in children exhibited nuclear estrogen and progesterone receptors. J Bone Miner Res.

[15] Smith EP, Boyd J, Frank GR (1994). Estrogen resistance caused by a mutation in the estrogen-receptor gene in a man. N Engl J Med.

[16] Ivanova J, Doukova P, Boyanov M, Popivanov P (2007). PvuII and XbaI polymorphisms of the estrogen receptor gene and bone mineral density in a Bulgarian population sample. Hormones.

[17] Wu J, Qiu Y, Zhang L, Sun Q, Qiu X, He Y (2006). Association of estrogen receptor gene polymorphisms with susceptibility to adolescent idiopathic scoliosis. Spine.

[18] Tang NL, Yeung HY, Lee KM, Hung VW, Cheung CS, Ng BK, Kwok R, Guo X, Qin L, Cheng JC (2006). A relook into the association of the estrogen receptor [alpha] gene (PvuII, XbaI) and adolescent idiopathic scoliosis:a study of 540 Chinese cases. Spine.

[19] Zhao D, Qiu GX, Wang YP, Zhang JG, Shen JX, Wu ZH (2009). Association between adolescent idiopathic scoliosis with double curve and polymorphisms of calmodulin1 gene/estrogen receptor-αgene. Orthop Surg.

[20] Takahashi Y, Matsumoto M, Karasugi T, Watanabe K, Chiba K, Kawakami N, Tsuji T, Uno K, Suzuki T, Ito M, Sudo H, Minami S, Kotani T, Kono K, Yanagida H, Taneichi H, Takahashi A, Toyama Y, Ikegawa S (2011). Replication study of the association between adolescent idiopathic scoliosis and two estrogen receptor genes. J Orthop Res.

[21] Cheng JC, Tang NL, Yeung HY, Miller N (2007). Genetic association of complex traits:using idiopathic scoliosis as an example. Clin Orthop Relat Res.

